# Who decides? Exploring decisional dynamics for periviable resuscitation among diverse family structures

**DOI:** 10.1038/s41372-025-02290-5

**Published:** 2025-04-16

**Authors:** Erika R. Cheng, Shelley M. Hoffman, Victoria Schultz, Naomi Castellon-Perez, Hannah Witting, Carli King, Brownsyne Tucker Edmonds

**Affiliations:** Indiana University School of Medicine, Indiana University, Indianapolis, IN USA

**Keywords:** Medical ethics, Health services

## Abstract

**Objective:**

To investigate conflict resolution and decisional authority among diverse family structures in periviable resuscitation decision-making.

**Methods:**

We recruited 60 parent dyads, including 30 with prior periviable delivery experience and 30 first-time expecting dyads between 22 and 26 weeks gestation. Our diverse sample included heterosexual and same-sex partnerships, married and unmarried partners. Virtual interviews explored decision-making, engagement, and conflict resolution.

**Results:**

Four themes emerged: “Mom is the priority,” highlighting maternal decisional authority; “partner involvement is crucial,” emphasizing engagement; “parents prioritize who will be caring for the child,” indicating caregiver considerations; and “parents want empathy and support,” underscoring emotional needs.

**Conclusions:**

Findings reveal maternal priority in decision-making, the importance of partner involvement, and a challenges with lacking inclusive legal and ethical guidance for non-heteronormative families. The study highlights the need for shared decision-making that considers family structures, legal aspects, and emotional complexities to enhance inclusive, informed decision-making processes for parents facing periviable delivery.

## Introduction

While medical innovation and discovery have increased survival rates for periviable neonates [1], early delivery before the third trimester remains a significant contributor to neonatal and infant mortality and often results in long-term disabilities for survivors [2]. These outcomes place substantial emotional, mental, and financial strain on families [3]. In the event of a potential periviable delivery, parents face the critical decision between palliation or resuscitation. Furthermore, survival is not guaranteed with resuscitation and may result in mild to profound lifelong neurodevelopmental disabilities. It is crucial that parents are adequately prepared to make these decisions, as this preparation can enhance decision satisfaction and reduce the risk of postpartum mental health disorders [4, 5].

The American Academy of Pediatrics advocates for shared decision-making (SDM) as the ideal approach for these scenarios [6]. However, current SDM practices in neonatal care often prioritize the pregnant patient’s perspective, overlooking the input of coparents, which can lead to discord and lower decision satisfaction [7]. The issue is further complicated in non-heteronormative family structures, where traditional assumptions about decisional authority based on heteronormativity can introduce systemic bias. Although legal progress, such as the 2017 revision of the Uniform Parentage Act [8], has recognized parental rights for diverse families, implementation remains limited and decisional authority for coparents and non-heteronormative families remains vague, leaving providers and families with lacking ethical and legal guidance to navigate this space.

To address these challenges, we conducted qualitative research exploring the perspectives of pregnant individuals and their partners, in diverse family structures, on the topics of conflict resolution, decisional authority, and partner involvement in periviable delivery decision-making. We interviewed both expectant parents and those who previously experienced periviable delivery, believing that insights from both perspectives are vital for guiding providers in facilitating successful, informed, and collaborative decision-making. Recognizing these issues for both heteronormative and non-heteronormative family structures has the potential to refine models for shared decision-making and healthcare delivery in periviability.

## Materials and methods

### Study design and participant recruitment

Approval for all study procedures was obtained from the Indiana University Institutional Review Board (IRB #2011857800) and was performed in accordance with the Declaration of Helsinki. Our goal was to assemble a diverse and representative cohort, including parent dyads who had experienced a periviable delivery (‘Experienced Dyads’) and those who were first-time expecting parents between 22 and 26 weeks’ gestation at the time of their scheduled interview (‘Prospective Dyads’). Both parents were required to participate, engage in separate interviews, speak English or Spanish, and be at least 18 years old.

Parent dyads were defined as having a birthing parent (i.e., the person who delivered a periviable baby or was currently pregnant within the periviable window) and a partner, whether biologically related or not, who intended to coparent the child. We intentionally recruited a diverse array of cohabiting partnerships, encompassing both heterosexual and same-sex partnerships, as well as married and unmarried partners. Romantic involvement was not required; rather, we focused on partners who ‘intended’ to co-parent with the birthing parent at the time of delivery, which could include a friend or relative, such as a grandparent or sibling.

We aimed to recruit 30 Experienced dyads and 30 Prospective dyads, totaling 120 participants. Recruitment efforts included social media platforms like Facebook and Reddit, where we distributed study fliers and screening surveys. We targeted groups focusing on preterm birth, periviable birth and preterm loss for Experienced dyads, and topics concerning first-expecting parents for Prospective dyads. To ensure diversity, we specifically reached out to LGBTQ + , non-binary, Hispanic, and blended family fertility, pregnancy and parenting groups. We obtained permission prior to posting in these groups, with biweekly posts on Facebook and weekly posts on Reddit. In total, we contacted 201 social media groups, including 65 LGBTQ+ Parent Groups and 28 Diverse Family Groups.

Beyond social media, we distributed flyers in coffee shops, children’s toy and clothing stores, and clinics, as well as had a booth at a LGBTQ+ Pride Fest and an OutCare Gala. The study was advertised in local newsletters and research volunteer registries. Additionally, we identified Experienced parents from our prior work [9–12] and through medical records.

Interested candidates, after viewing the advertisement online, completed a brief screening questionnaire via a provided link. Those initially deemed eligible were asked to provide contact information and were followed up by a research assistant who confirmed their eligibility with additional screening questions. If eligible, they were asked to provide their partner’s contact information, who was then also screened. Once eligibility was confirmed, separate 1-h virtual interviews were scheduled. Recruitment took place from December 2021 to April 2023.

### Interview structure and development

The interview was structured into four parts. Recognizing that obstetric decisions are often made between provider and the birthing person, we aimed to explore parents’ perceptions of the non-birthing parent’s role in neonatal treatment decisions (e.g., resuscitation or comfort care). Prospective dyads were presented with a hypothetical scenario of being admitted for periviable complications and asked how, if at all, the non-birthing partner should be involved. Conversely, Experienced dyads answered questions based on their experience with their respective periviable delivery, such as whether the non-birthing partner was involved, if they disagreed on a treatment option, etc. To explore the non-birthing partner’s role and decisional authority, questions were intentionally framed around the interviewee. For example, when interviewing the birthing person, questions were phrased in terms how they would want their *non-birthing* partner involved. Conversely, when interviewing the non-birthing partner, questions centered on *their* actual or desired level of involvement.

The second part of the interview consisted of four scenarios with yes/no responses to assess whether a partner’s decisional authority or ‘final say’ should be influenced by factors such as marital status, biological relationship to the child, involvement in the pregnancy, and intention to be involved in the child’s life.

The third section featured a clinical case vignette portraying a married, heterosexual couple hospitalized at 22 weeks’ gestation for a threatened periviable delivery. In the scenario, the birthing person, Mrs. H, strongly favors resuscitation while both the biological father, Mr. K, and healthcare team advocate for comfort care. Participants were asked to consider how Mrs. H and Mr. K should navigate this disagreement, determine who should have ultimate decisional authority, and discuss whether the healthcare team should play a role in conflict resolution. The scenario was then reversed, with Mrs. H and the healthcare team agreeing on comfort care, while Mr. K insists on resuscitation. This section concluded with seven additional scenarios exploring decisional authority in non-traditional family structures where H and K represent diverse familial contexts such as surrogacy (gestational carrier), adoption, same-sex relationships, and unmarried co-parents.

The final section of the interview focused on participant demographics. Verbal, informed consent was obtained by a trained research assistant and all interviews were audio recorded. Participants received a $50 gift card ($100 per dyad) upon interview completion.

### Analysis

Survey responses were summarized using descriptive statistics. Interviews were audio-recorded, transcribed verbatim, deidentified, and carefully checked for accuracy. A codebook, incorporating codes derived from the interview questions, was developed by study personnel. All 120 interviews were coded, with consensus achieved through double coding by four research assistants. Quantitative data were securely stored in a REDCap (Research Electronic Data Capture) database and used for frequencies and descriptive analysis.

## Results

### Demographics

Participant demographics are summarized in Table 1. The majority of parents identified as White (Experienced = 68%, Prospective = 85%), non-Hispanic (90%), and heterosexual (Experienced = 95%, Prospective = 83.3%). A higher proportion of Prospective parents were in single, coparenting partnerships (86.7%) compared to their married, Experienced counterparts (81.7%). While there was more LGBTQ+ representation in the Prospective group, overall representation was limited. In total, we had 10 parenting dyads (20 participants) from the LGBTQ+ community.

**Table 1:** Demographic characteristics of prospective and experienced parents, *N* = 120.

	Prospective	Experienced
**Mean Age (Range)**	30.02 ± 5.04	36.01 ± 6.86
	***n*** **(%)**	***n*** **(%)**
**Race**		
White	51 (85%)	41 (68%)
Black/African American	6 (10%)	11 (18.3)
Asian	2 (3.3%)	1 (1.7%)
Native Hawaiian, Pacific Islander	0	3 (5%)
American Indian, Alaskan Native	0	1 (1.7%)
Multiracial	3 (5%)	7 (11.7%)
Other/Prefer not to disclose	1 (1.7%)	0
**Ethnicity**		
Non-Hispanic/Latinx	54 (90%)	54 (90%)
Hispanic/Latinx	5 (8.3%)	4 (6.7%)
Prefer not to disclose	1 (1.7%)	2 (3.3%)
**Sex at Birth**		
Male	29 (48.3%)	26 (43.3%)
Female	31 (51.7%)	33 (55%)
Prefer not to disclose	0	1 (1.7%)
**Gender**		
Cis-gender male	28 (46.7%)	26 (43.3%)
Cis-gender female	29 (48.3%)	33 (55%)
Trans-gender male	1 (1.7%)	0
Trans-gender female	0	0
Non-binary	2 (3.3%)	0
Prefer not to disclose	0	1 (1.7%)
**Sexuality**		
Heterosexual	50 (83.3%)	57 (95%)
Gay	1 (1.7%)	0
Lesbian	1 (1.7%)	0
Bisexual	5 (8.3%)	2 (3.3%)
Other	2 (3.3%)	0
Prefer not to disclose	1 (1.7%)	1 (1.7%)
**Marital Status**		
Single, never married	52 (86.7%)	9 (15%)
Married/partnered	8 (13.3%)	49 (81.7%)
Divorced/separated	0	2 (3.3%)

Presents demographic characteristics of both prospective and experienced parent participants.

### The role of partners in periviable decision making

Overall, participants overwhelmingly agreed that non-birthing partners should be actively involved in the decision-making process and receive the same information from providers. They described their approach as a “unified” and “together” effort, emphasizing the importance of mutual support when faced with making neonatal treatment decisions.

*“Just as much as basically I would be included… I just want us to both have the exact same information to make the decision.” – Prospective Birthing Parent, Case ID #382*

*“How can we walk through it together in the best scenario possible? Of course, it’s a, in a sense, big-lose little-win situation kind of talk, but like I said before, it’s always best to be clear and upfront and honest with each other.” – Experienced Partner, Case ID #1447*

While participants unanimously agreed on the importance of joint involvement, the extent of this involvement varied. Many Prospective partners believed that the ultimate decision should rest with the birthing person, using phrases like “her body” and “her choice”. They expressed a readiness to support and “advocate” for the birthing person’s preferences.

*“I think if she has her mind made up either way, I would be more inclined to lean that way, regardless of what I thought.” – Prospective Partner, Case ID #1238*

*“I don’t want to take away bodily autonomy… she’s going through a lot… and I don’t want to take that away from her.” – Prospective Partner, Case ID #633*

Several of the Experienced non-birthing partners reported positive interactions with healthcare providers, feeling actively included in discussions about neonatal resuscitation. Providers were noted for facilitating inclusive communication by saving “big discussions” for times when both parents were present, using inclusive language such as “you both”, engaging with and maintaining eye contact with both parents, inquiring if there were any questions, and ensuring that updates were communicated to both parents before and after delivery.

*“They* [the healthcare team] *always made sure that I was there… They always included me in the choices… They would ask me my opinions and it made me feel like I was part of helping make choices.”— Experienced Partner, Case ID #1460*

However, some partners described specific moments when they felt excluded by providers. One participant shared how, despite being initially included in discussions, he felt marginalized and excluded from conversations after pregnancy was declared nonviable.

***“****How you face, the way you position your legs, the way that you’re positioning your head or you’re turning your shoulder, and when they would come in and they would talk to [partner name], it would be they were making me stand behind them no matter where I was at. Obviously, I was bedside, right?”— Experienced Partner, Case ID #693*

### Navigating disagreement

All Experienced parents reported unanimity in their decisions regarding resuscitation or palliation. Consequently, participants from both groups outlined strategies for navigating hypothetical disagreements. These strategies included seeking additional information from online resources and healthcare providers, as well as involving a “third party”, such as a therapist, religious/spiritual leaders, or counselors, to help mediate disputes. Participants underscored the significance of compromise, with some expressing willingness to set aside their own preferences for the well-being of their or the relationship. They also expressed readiness to prioritize their partner’s feelings and decisions.

*“I think we would just… agree and be realistic on the situation. And while some things might be not so, how do I say, agreeable by one party, I think in these medical situations, you just have to compromise.”— Prospective Partner, Case ID #296*

A recurring theme throughout the discussions was the acknowledgment of the birthing parent’s unique position, decision-making authority, and willingness of their partners to ultimately defer to their judgment and needs.

*“I honestly would’ve pulled that card if we were to disagree. That sounds horrible… I would’ve done pretty much anything at that point…” — Experienced Birthing Parent, Case ID #847*

*“I do believe he would defer to my judgment first and foremost.” — Prospective Birthing Parent, Case ID #278*

*“She is the one that is pregnant and growing a child… I feel comfortable deferring to her decision-making in that situation.” — Prospective Partner, Case ID #525*

*“If she wanted to resuscitate or not, I would’ve just supported her.” — Experienced Partner, Case ID #1672*

### The impact of marriage, biology, and involvement on partners’ decisional authority

Overall, marriage was deemed the least influential factor in determining a non-birthing partner’s decisional authority in neonatal treatment decision-making (35.8%), although Experienced parent dyads were more likely to favor marriage than their Prospective counterparts (41.7% vs. 30%) (Table 2). In contrast, both parent groups agreed that a partner’s intention to be involved in a child’s life had the greatest impact on decisional authority (72.5%). Within dyads, Prospective birthing parents prioritized involvement in the child’s life (76.7%) over involvement in pregnancy (60%). In contrast, their partners (the non-birthing parent) placed greater emphasis on being involved in pregnancy (70%) than in the child’s life (63.3%). Similarly, Experienced birthing parents emphasized both involvement in pregnancy (76.7%) and in the child’s life (83.3%) more than their partners did (60% and 66.7%, respectively).

**Table 2:** Parents’ considerations for marriage, biology, and involvement as factors that should affect a partner’s decisional authority in periviable delivery decision making.

	Overall			Prospective			Experienced		
*Should decisional authority be affected:*	Prospective (*n* = 60)	Experienced (*n* = 60)	*p*-value	Birthing parent (*n* = 30)	Partner (*n* = 30)	p-value	Birthing parent (*n* = 30)	Partner (*n* = 30)	*p*-value
If the partner is married to the pregnant person?	18 (30%)	25 (41.7%)	0.18	9 (30%)	9 (30%)	1.0	12 (40%)	13 (43.3%)	0.79
If the partner is biologically related to the child?	38 (63.3%)	40 (66.7%)	0.70	19 (63.3%)	19 (63.3%)	1.0	20 (66.7%)	20 (66.7%)	1.0
Depending on the amount of involvement the partner has had with the pregnant patient during the pregnancy?	39 (65%)	41 (68.33%)	0.60	18 (60%)	21 (70%)	0.42	23 (76.7%)	18 (60%)	0.22
Depending on the amount of involvement the partner intends to have in the child’s life?	42 (70%)	45 (75%)	0.35	23 (76.7%)	19 (63.3%)	0.26	25 (83.3%)	20 (66.7%)	0.28

Presents participants’ responses to four “yes/no” scenarios regarding factors that should influence a partner’s decisional authority - marital status, biological relationship to the child, involvement in the pregnancy, and intention to be involved in the child’s life.

### Parents’ perspectives of decisional authority in non-heteronormative scenarios

Table 3 displays parents’ perspectives of decisional authority when presented with seven scenarios involving non-heteronormative family structures. The majority of participants assigned decisional authority to the birthing parent (Ms. H) in scenarios involving a heterosexual couple, regardless of the relationship context, emphasizing that she is “the mother,” “it’s her baby,” “it’s her body,” and “she’ll be the one responsible for it.” Few parents were inclined to grant Mr. K any decisional authority, particularly if he was not intending to coparent (0%) or not the biological father (3.3%). Participants who elaborated on their reasoning for each scenario often expressed support for whichever parent advocated for resuscitation, regardless of their role or circumstance.

*“If both are equally involved… leaning towards, again, if everything else seems okay, then pursue resuscitation.” – Experienced Partner, Case ID #1824*

*“I think the mom because her goal is to give their baby a chance at life.” – Prospective Partner, Case ID #1140*

**Table 3:** Parents’ assignment of ultimate decisional authority in non-heteronormative family structures.

*Who should have the ultimate decisional authority in the following scenarios?*	Ms. H		Mr. K		Mr. & Mrs. O		Mr. & Mrs. B		Declined to answer	
	*P*	*E*	*P*	*E*	*P*	*E*	*P*	*E*	*P*	*E*
If Ms. H and Mr. K are not married, but Mr. K is planning to coparent?	58 (96.7%)	53 (88.3%)	0	5 (8.3%)					2 (3.3%)	2 (3.3%)
If Ms. H and Mr. K are not married and Mr. K is not planning to coparent?	60 (100%)	60 (100%)	0	0					0	0
If Ms. H and Mr. K are not married and Mr. K is not the biological father, but he intends to coparent?	58 (96.7%)	57 (95%)	0	2 (3.3%)					2 (3.3%)	1 (1.7%)
If Ms. H and Mr. K are the biological parents, but have selected Mr. and Mrs. O as adoptive parents?	14 (23.3%)	16 (26.7%)	0	0	46 (76.7%)	44 (73.3%)			0	0
If Ms. H is a gestational carrier (surrogate carrying their embryo) for Mr. and Mrs. B who are the biological parents?	6 (10.0%)	4 (6.7%)	0	0			53 (88.3%)	56 (93.3%)	1 (1.7%)	0
***Now consider if Mrs. H and Mr. K were instead Mrs. H and MRS. K, a married same-sex couple****.*	**Mrs. H**		**Mrs. K**						**Declined to answer**	
	***P***	***E***	***P***	***E***					***P***	***E***
Mrs. K is both the pregnant patient (carrier) and biologically related mother (egg donor)?	4 (6.7%)	1 (1.7%)	56 (93.3%)	58 (96.6%)					0	1 (1.7%)
Mrs. K is the pregnant patient (carrier) and Mrs. H is the egg donor/ biologically related mother?	20 (33.3%)	18 (30%)	38 (63.3%)	40 (66.7%)					2 (3.3%)	2 (3.3%)

^*^P Prospective parent, *E* Experienced parent. Note: some totals may not add to 100% due to rounding.

Presents parents’ perspectives of decisional authority for seven non-traditional family structure scenarios.

In non-heteronormative family scenarios, many parents sought legal guidance and referred to the person they felt had “legal responsibility” for the child. This contrasted with discussions about decision-making in heteronormative family structures, where the default was often the mother, and legal considerations were less frequently discussed. In scenarios involving adoption and surrogacy, participants consistently prioritized decisional authority to the individual(s) designated to care for the child after delivery. For instance, both Prospective parents (76.7%) and Experienced parents (73.3%) sided with Mr. and Mrs. O, the adoptive parents, even though Ms. H and Mr. K were the biological parents. Similarly, in the surrogacy scenario, approximately 90.8% of parents assigned decisional authority to the biological parents, Mr. and Mrs. B, whereas only 8.3% sided with Ms. H, the gestational carrier.

Participants expressed more divided opinions on decisional authority when presented with scenarios involving a same-sex couple.

An overwhelming majority of participants assigned decisional authority to Mrs. K when she was both the carrier and the egg donor (Experienced = 96.6%, Prospective = 93.3%). However, when Mrs. K’s partner, Mrs. H, was the biological parent, only 66.7% of Experienced parents and 63.3% of Prospective parents assigned decisional authority to Mrs. K., with some saying that “it’s [Mrs. K’s] body” and others “it’s [Mrs. H’s] baby.

When we considered the responses from individuals in non-heteronormative relationships, we found that 95% (19/20) sided with Mrs. K when she was both the carrier and donor, and 75% (15/20) when she the carrier and not the biological parent.

## Discussion

This study qualitatively explored parents’ perspectives of conflict resolution, decisional authority, and partner involvement in periviable delivery decision-making. Overall, participants consistently emphasized the importance of involving both partners in the decision-making process, with a strong focus on mutual support and the need for shared information. However, the extent of this involvement varied based on factors such as marital status, biological connection, and intended involvement in the child’s life.

Our findings suggest that marriage status is one of the least significant factors in determining a non-birthing partner’s decisional authority, suggesting that legal or social constructs of marital status may be less relevant in high-stakes medical decision-making contexts. Instead, the intention to be involved in the child’s life emerged as the most critical factor, indicating that emotional and practical commitment to the child holds greater weight in these decisions. Notably, our own state’s statute only endows decisional standing to fathers who are married to the birthing person [13]. Interestingly, prospective parents prioritized involvement during pregnancy, experienced parents placed more emphasis on involvement in the child’s life. This shift in perspective may reflect the impact of lived experiences in parenthood, where long-term responsibilities become more apparent. Additional complexities arise when biology and intended parentage are uncoupled, such as in adoption, surrogacy, or assisted reproduction. These insights call for a more expansive set of considerations than currently accounted for in existing legal and ethical decision-making models.

Our data suggest that while there is strong support for the involvement of non-birthing partners, there remains a pervasive belief that the birthing parent should have the final say, particularly in situations where there is disagreement. The tendency to default to the birthing parent for decisional authority, regardless of the family structure, reflects widely held societal norms and assumptions about maternal authority in caregiving [4]. It also highlights potential tensions that must be addressed in efforts to promote more family-centered models of shared decision-making. Indeed, while prioritizing the birthing person aligns with existing models of SDM [2, 3] it prompts reflection on how to incorporate important others into decision-making should the pregnant person desire such support, while simultaneously recognizing the unique significance of the birthing person’s role. Even in scenarios of conflict, participants expressed that the birthing person should be central to the decision-making process, which emphasizes their autonomy and reflects a deep regard for their agency and autonomy in determining the course of action.

Variability in responses to our vignette scenarios suggests that healthcare providers may need to adopt more nuanced and inclusive communication strategies that incorporate partners, account for non-traditional family structures, and help families navigate disagreement. Experienced partners generally reported positive interactions with healthcare providers, but some noted instances of exclusion from the decision-making process. Strategies for navigating disagreements included seeking additional information and involving third parties for mediation. Providers should be trained to recognize and address the diverse family structures and dynamics that may encounter, ensuring that all parties feel heard and respected in the decision-making process. This is particularly important in non-heteronormative scenarios, where traditional assumptions about decisional authority may not apply.

Although some prior work has acknowledged the role of “important others” in making periviable delivery decisions [4], most existing literature has focused on heterosexual partnerships. Our findings underscore the importance of recognizing and accommodating LGBTQ+ and other diverse family structures in periviable decision-making. In our cohort, decisional authority considerations in non-heteronormative scenarios, including same-sex partnerships, adoption, and surrogacy, demonstrated distinct patterns. Here, participants often deferred to legal guidance and the concept of legal responsibility for the child. Such guidance is largely lacking. This reliance on legal frameworks, and the lack thereof, underscores the need for clear and inclusive legal and ethical guidance that addresses the unique challenges faced by diverse families in medical decision-making.

### Implications for SDM

The study’s findings challenge the existing framework of SDM in periviable care, which predominantly centers on the pregnant individual to the exclusion of desired support persons. Feminist ethnics underscore the importance of relational dynamics in maternal decision-making [14]. To that end, decision making models that include partners or loved ones may enhance, rather than undermine, autonomous decision making in these settings. To optimize decisional outcomes, a paradigm shift is necessary, acknowledging the dynamic interplay of family structures, legal considerations, and emotional needs. Integrating these aspects into the SDM framework can pave the way for a more inclusive, informed, and supportive decision-making process for all parents facing periviable situations.

### Limitations and future directions

While this study provides valuable insights, certain limitations should be acknowledged. Despite our attempts to recruit a diverse sample, our participants were predominantly white, heterosexual and non-Hispanic, which raises questions about the generalizability. The cohort was also drawn from Indianapolis, Indiana, a region that tends to be more socially conservative. Furthermore, because none of the Experienced dyads disagreed on a neonatal treatment decision at the time of their deliveries, we were unable to learn how parents navigated disagreement, but rather had to ask them from a hypothetical perspective. Our work also lacks the perspectives of parent dyads with experience in surrogacy or adoption in the setting of periviable delivery. Future research should strive for greater diversity and experiences in participant representation.

Additionally, it is important to consider the legal context during the study period (2021-2023), when Indiana’s legislature passed and implemented an abortion ban that may have influenced participants’ perspectives on periviable decision-making and could have heightened the emphasis on the birthing parent’s decisional authority. Future research should include participants from various geographic and cultural backgrounds and consider the impact of local laws and policies to enhance the generalizability of the results. Moreover, exploring the perspectives of healthcare providers and their role in facilitating inclusive decision-making could offer a comprehensive understanding of the challenges and opportunities in periviable care.

In conclusion, the study contributes vital perspectives on decision-making in periviable care, advocating for an inclusive and nuanced approach that considers diverse family structures, prioritizes involvement, and recognizes the importance of empathy and support in healthcare interactions. The implications extend beyond individual decisional scenarios to reshape the broader landscape of shared decision-making in periviable care.

## Data Availability

The datasets generated during and/or analyzed during the current study are available from the corresponding author on reasonable request.
